# Supplementary material for: Mapping the evolution of fertility support policies in China: A content and instrumental analysis
Source: PLoS One. 2025 Oct 9;20(10):e0332137. doi: 10.1371/journal.pone.0332137 (PMC12510515; doi:10.1371/journal.pone.0332137)
Supplement: S1 Appendix — (ZIP) [file pone.0332137.s001.zip › S1 Appendix. 226 original policy documents/131-卫生部关于印发《中国妇幼卫生事业发展“九五”规划和2010年目标纲要》的通知(FBM-CLI-4-33038).doc]

卫生部关于印发《中国妇幼卫生事业发展“九五”规划和２０１０年目标纲要》的通知

**发布部门：** [卫生部(已撤销)](https://www.pkulaw.com/law/chl?Aggs.IssueDepartment=60222) [机构沿革](javascript:void(0))

**发文字号：**卫妇发[1996]第4号

**发布日期：**1996.11.05

**实施日期：**1996.11.05

**时效性：** [现行有效](https://www.pkulaw.com/law/chl?Aggs.TimelinessDic=01)

**效力级别：** [部门工作文件](https://www.pkulaw.com/law/chl?Aggs.EffectivenessDic=XE0303)

**法规类别：** [妇幼卫生](https://www.pkulaw.com/law/chl?Aggs.Category=09622)

卫生部关于印发《中国妇幼卫生事业
发展“九五”规划和２０１０年目标纲要》的通知
（１９９６年１１月５日　卫妇发〔１９９６〕第４号）

各省、自治区、直辖市卫生厅（局）、计划单列市卫生局、新疆生产建设兵团卫生局：
　　为了贯彻落实中共中央十四届五中全会《关于制定国民经济社会发展２０００年目标和２０１０年远景目标设想》提出的“搞好优生优育、提高人口质量”的要求，全面实现《九十年代中国儿童发展规划纲要》和《[中国妇女发展纲要](https://www.pkulaw.com/chl/33670a50094f0c40bdfb.html)》中规定的妇女、儿童卫生保健目标，我部组织制定了《中国妇幼卫生事业发展“九五”规划和２０１０年目标纲要》，现印发给你们。请结合各地实际情况认真组织实施，并将实施情况及时反馈我部妇幼卫生司。

附件：中国妇幼卫生事业发展“九五”规划和２０１０年目标纲要

　　一、前言
　　１９９６－２０１０年是我国改革开放和社会主义现代化建设承前启后，继往开来的重要时期。未来的１５年，我国社会和经济的发展为妇幼卫生事业发展拓展了视野和空间，提供了不可多得的历史机遇；我国卫生事业发展总目标和基本战略的确定，也对妇幼卫生事业的发展提出了更高的要求。为此，迈向２１世纪的妇幼卫生事业必须把握机遇，全面规划，进一步发展和壮大。《中国妇幼卫生事业发展“九五”规划和２０１０年目标纲要》（简称妇幼卫生规划及纲要）提出了一个中长期的妇幼卫生事业发展目标、指导方针、主要任务和策略措施，重点放在“九五”时期，同时对下世纪初的发展目标提出远景设想，以保持战略的连续性。

　　二、背景：
　　（一）“八五”期间，全国妇幼卫生系统坚持实事求是的作风、坚持妇幼卫生工作方针和全心全意为妇女儿童健康服务的宗旨，紧紧把握社会需求，主动适应市场经济规律，以妇幼卫生合作项目为突破口，以保健与临床相结合和创建爱婴医院为契机，积极稳妥地进行改革；充分利用有限的妇幼卫生资源，调动社会各方面积极性，努力提高妇幼卫生服务能力；在机构建设、法制建设、建立新的服务模式、完善运行机制、实行保健保偿、加强科学管理、强化政府领导、发挥专家作用、专业建设与培训、应用科研等方面都有了长足的发展或进行了大胆而有益的探索。以降低孕产妇死亡率为重点，加强高危孕产妇管理，提高住院分娩率和产科质量；以降低婴儿和５岁以下儿童死亡率为核心，加强体弱儿童管理，推广急性呼吸道感染和腹泻病例管理、新生儿复苏、口服补液疗法等适宜技术；按生命的准备、生命的保护和晚年生活质量三个阶段对妇女儿童分阶段进行系列保健。“八五”期间，除新生儿破伤风防治取得一定进展外，其它工作均已如期达到《九十年代中国儿童发展规划纲要》（以下简称《儿童发展纲要》）确定的１９９５年中期目标。
　　（二）面临的困难及原因：
　　１、实现２０００年《儿童发展纲要》和《[中国妇女发展纲要](https://www.pkulaw.com/chl/33670a50094f0c40bdfb.html)》（以下简称[《妇女发展纲要》](https://www.pkulaw.com/chl/33670a50094f0c40bdfb.html)）提出的各项指标任务艰巨。据监测统计资料分析，１９９０年－１９９５年全国５岁以下儿童死亡的前５位死因仍无明显变化，肺炎仍是引起５岁以下儿童死亡的首位死因，表明我国儿童可控制性死亡仍没有较好的解决；我国新生儿死亡率下降十分缓慢，近年来新生儿疾病占５岁以下儿童死亡的百分比呈上升趋势，新生儿死亡严重影响着婴儿和５岁以下儿童死亡率的降低；１９９０－１９９５年，产科出血一直是我国孕产妇死亡的第一位死因，占孕产妇总死亡比波动在５０％左右，实现到２０００年因产科出血引起死亡减少一半的任务十分艰难；５岁以下儿童中、重度营养不良患病率受社会和经济发展的直接影响，在“九五”期末与１９９０年比尚有再下降２７％的繁重任务；我国新生儿破伤风高发地区地广人稀，交通不便，卫生条件差，开展新法接生和破伤风类毒素免疫接种工作难度大；全面开展婚前医学检查、产前诊断和对先天性疾病的筛查工作的各项措施还尚未完全落到实处，到２０００年将先天性病残儿发病率减少一半的目标难于实现；我国妇幼卫生工作发展不平衡，广大农村特别是边远贫困地区还牵制着全国各项平均指标的降低。
　　２、随着经济、社会的发展，人民生活水平的提高和医学模式的改变，一方面妇女儿童保健需求增加，期望能获得更多的生理、心理等保健服务；另一方面随着城市化、工业化进程的加速，环境、生态等新的公共卫生问题的出现，以及由于生活环境、生活方式、精神因素、劳动卫生等引起的疾病逐年增加，诸多生殖健康问题急待解决。近年来，流动人口中妇女、儿童保健问题也很严重，妇幼卫生面临新的挑战。
　　３、妇幼卫生工作在经济和社会发展以及卫生事业发展战略中的地位，还没有形成各级领导和全社会的共识。部分地方领导还没有纳入议事日程，成为政府行为；妇幼卫生政策保障滞后的局面未得到改变，经费长期投入不足同妇幼卫生工作任务的迅速增加不相适应。
　　４、虽然近几年妇幼卫生保健机构有了一定发展，但同社会发展仍不相适应。个别省、部分市（地）、县至今尚无妇幼保健机构或妇幼卫生行政管理部门，有的虽有机构但未达到国家基本标准。业务用房不足，危房多，设备严重短缺，技术水平不高，整体功能不足，服务能力差，管理手段和思维观念落后等已成为妇幼卫生部门的普遍问题。

　　三、指导思想和指导方针
　　“九五”期间和下世纪初我国妇幼卫生事业发展指导思想是：以建设有中国特色的社会主义理论为指导，继续坚持妇幼卫生工作方针，明确政府职责，保证妇幼卫生工作与社会经济同步发展，强化卫生执法，以人、人的健康和人群为中心，以最大限度满足妇女儿童基本保健需求为导向，深化改革，改变服务模式，主动适应社会主义市场经济和医学模式转变的发展趋势，为落实我国人口政策和实现政府承诺服务。
　　今后十五年应坚持以下指导方针：（一）坚持为实现两个《纲要》和“２０００年人人享有卫生保健”的战略目标服务。（二）坚持为“控制人口数量，提高人口素质”的基本国策服务。（三）坚持贯彻落实《[中华人民共和国母婴保健法](https://www.pkulaw.com/chl/ea633acd04298a40bdfb.html)》，依法管理并提供母婴保健服务。（四）坚持以保健为中心，保健和临床相结合，面向基层，面向群体的妇幼卫生工作指导方针。（五）坚持从实际出发，因地制宜，分类指导，实事求是的工作原则。（六）坚持树立大卫生观念，强化政府行为，注重多部门支持与协作，充分利用一切妇幼卫生资源，动员社会各方面积极支持和参与，促进妇幼卫生事业的发展。（七）坚持妇幼卫生事业与社会经济及整个卫生事业的协调发展。

　　四、战略目标与主要指标
　　（一）总目标
　　“九五”期间妇幼卫生工作的总目标是：努力实现我国政府承诺的《儿童发展纲要》和[《妇女发展纲要》](https://www.pkulaw.com/chl/33670a50094f0c40bdfb.html)中的各项妇幼卫生目标；建立具有中国特色的全心全意为广大妇女儿童服务的社会主义妇幼卫生服务体系及运行机制；逐步建立起有利于我国广大妇女儿童健康的社会保障制度；依法规范妇幼卫生的管理和服务，逐步完成妇幼卫生由行政管理向依法行业管理的转变；使妇幼卫生事业同国民经济、社会发展和人民生活达到小康水平相适应，同全面提高人口素质的人口政策相适应。
　　（二）主要健康指标
　　１、到２０００年全国婴儿死亡率达到３４‰以下，实现在１９９０年的基础上降低１／３的目标；２０１０年在２０００年基础上再降低１／４。
　　２、到２０００年５岁以下儿童死亡率达到４１‰，实现在１９９０年基础上降低１／３；２０１０年在２０００年基础上再降低１／４。
　　３、到２０００年孕产妇死亡率达到４７／１０万以下，实现在１９９０年的基础上降低１／２的目标；２０１０年在２０００年基础上再降低１／３。
　　（三）具体工作目标
　　到２０００年，具体工作要实现以下目标：
　　１、５岁以下儿童保健覆盖率以县为单位达到８５％。
　　２、９０％以上的儿童急性呼吸道感染实行病例管理，５岁以下儿童因急性呼吸道感染死亡人数比１９９０年减少１／３。
　　３、５岁以下儿童因腹泻死亡的人数比１９９０年减少１／２，腹泻患病人数减少２５％。
　　４、母乳喂养率以省为单位达到８０％，县及县以上设有产、儿科床位的医疗保健机构都成为爱婴医院。
　　５、５岁以下儿童中、重度营养不良患病率在１９９０年的基础上降低１／２。
　　６、孕产妇保健覆盖率以县为单位达到８５％。
　　７、９５％以上高危妊娠及产科紧急情况能够及时处理或转诊，高危孕产妇住院分娩达９５％以上。农村孕产妇住院分娩率达６０％以上，因产科出血引起的死亡减少一半。
　　８、因新生儿窒息死亡减少１／３。
　　９、农村新法接生率达９５％，新生儿破伤风发病率以县为单位降低到１／１０００活产儿以下。
　　１０、８５％以上的县具备婚前医学检查和产前诊断条件，并开展优生咨询服务；婚前医学检查率以省为单位达到８５％；８０％的省具备规范的、管理有序的对先天性疾病筛查工作的网络，降低先天性病残儿发生率。
　　１１、母婴保健保偿、合作医疗等多种形式的妇幼保健保障制度的覆盖率以省为单位达到６０％。
　　１２、以家庭为单位妇幼保健知识普及率达到７０％。
　　１３、产科急救、产科技能、小儿急性呼吸道感染病例管理、口服补液疗法等适宜技术培训的覆盖率，乡级妇幼保健人员达到９５％，村级达到８０％。
　　以上各项工作指标，２０００年－２０１０年期间应继续巩固、提高。

　　五、主要对策和措施
　　（一）明确和强化政府职责，进一步加强对妇幼卫生事业的重视和领导。妇女、儿童的健康是人类生存和发展的要素，是我国国民经济和社会发展的重要内容，妇幼卫生工作是社会保障体系的重要组成部分，对于促进和保障社会经济发展起着重要作用。实现我国政府承诺的《儿童发展纲要》和[《妇女发展纲要》](https://www.pkulaw.com/chl/33670a50094f0c40bdfb.html)的目标是各级政府的责任，应遵循政府负责、部门配合、群众参与、法制保障的原则，切实加强对妇幼卫生工作的领导，把妇女儿童的健康纳入经济和社会发展的“九五”规划，制订发展政策，列入议事日程，落实责任管理，使“儿童优先、母亲安全”成为全社会的共识。
　　（二）充分认识妇幼卫生的基础作用和战略地位
　　各级卫生行政部门要充分认识妇幼卫生工作在促进经济建设、落实人口政策、提高人口素质、实现初级卫生保健中的基础作用和战略地位。妇女、儿童占总人口的三分之二，是最需要得到健康保护的脆弱人群。各级卫生部门应努力当好政府参谋，合理调整妇幼卫生在卫生事业资源分配中的份额，摆正妇幼保健在卫生工作中的位置。
　　“九五”期间，各级卫生行政部门应加强妇幼卫生行政机构和执法队伍的建设，转变职能，提高工作效率，逐步实现由微观向宏观、由部门向行业、由行政向法制管理的过渡，进一步实现妇幼卫生的全行业管理。
　　（三）落实人口政策，努力提高人口素质
　　“九五”期间，各级妇幼保健机构要依法开展生殖健康的各项服务内容，以生殖保健为中心，认真开展女童保健、青春期保健、婚前保健、优生咨询、围产保健、儿童保健，做到少生、优生、优育。努力减少残疾儿的出生，提高出生人口的素质。
　　（四）以农村为重点，切实加强妇幼保健网的建设
　　实现妇幼卫生战略目标，关键在于抓好农村，要切实加强县、乡、村三级妇幼卫生网的建设，保证卫生政策和资源向边远贫困地区倾斜。
　　１、省、市（地）级妇幼保健机构要强化业务管理职能和监督指导能力，对本地区的妇幼保健工作进行全面的组织实施、技术指导与培训。到２０００年８５％的省、市（地）级妇幼保健机构达到《妇幼保健机构评审标准》中规定的相应级别的甲等或乙等妇幼保健院标准，并具备有关的医学遗传筛查等专项技术服务能力。
　　２、到２０００年，全国基本完成县级妇幼保健机构的建设，８５％的县级妇幼保健机构达到《妇幼保健机构评审标准》中规定的一级甲等妇幼保健院建设标准，增强预防意识、群体意识、服务意识，具备《[母婴保健法](https://www.pkulaw.com/chl/ea633acd04298a40bdfb.html)》中规定的有关婚前医学检查等专项技术服务能力，成为本地执法服务指导和人员培训中心。
　　３、农村乡镇卫生院要重点加强产、儿科建设，努力提高住院分娩率和产科质量，降低产后出血和新生儿窒息死亡率。
　　４、村卫生室要做好孕产妇保健、儿童保健、信息统计、健康教育等妇幼卫生基础工作，努力提高保健覆盖率、系统管理率和家庭妇幼保健知识普及率。
　　５、增加对乡、村妇幼卫生的投入，落实基层妇幼卫生技术人员的有关政策，合理解决报酬，为稳定基层妇幼卫生队伍创造有利条件。
　　（五）推进妇幼卫生法制建设，加大妇幼卫生执法力度
　　在加强妇幼卫生宏观调控中，要重视妇幼卫生法制建设，研究和制订有关法规政策、规章制度和业务技术标准。建立完善妇幼卫生监督与执法体系，加大执法力度。
　　１、“九五”期间各地要依据《[母婴保健法](https://www.pkulaw.com/chl/ea633acd04298a40bdfb.html)》，制订地方配套法规，建立健全执法机构，充实加强执法、监督队伍，加快执法队伍培训，进一步加强《[母婴保健法](https://www.pkulaw.com/chl/ea633acd04298a40bdfb.html)》的宣传，增强全社会的法制观念和保健意识。
　　２、严肃认真履行法定职责，对婚前医学检查、助产技术、产前诊断、遗传病诊断、终止妊娠、结扎手术以及从事家庭接生等法律规定的专项技术，依法实行行业管理；依法实行规范服务。卫生行政部门在依法实施行业管理中，要主动协调有关部门，争取各部门和社会支持，加大执法力度。
　　（六）增加政府投入，扩大妇幼卫生资源
　　我国现阶段卫生资源以国家投入为主，同时多渠道、多层次筹集。各级政府要调整卫生投入的结构与政策，根据各级妇幼保健机构的实际情况，研究制定经费补助办法和管理办法，加大对妇幼卫生的投入，确保妇幼卫生事业经费增长要高于卫生事业费的年增长速度。
　　１、国家和各级政府应在《[母婴保健法](https://www.pkulaw.com/chl/ea633acd04298a40bdfb.html)》执法工作中设立专项经费，并建立相应专款，用于贫困地区实现两个《纲要》目标的妇幼卫生扶助经费，加快改变贫困地区妇幼卫生薄弱状况。
　　２、要保证妇幼保健机构的全额拨款，逐步提高妇幼保健机构的财政补助水平。继续拓宽旨在促进妇女儿童发展的多种社会筹资渠道，提倡和鼓励企事业单位、社会团体和公民个人捐资、集资，建立基金，支持妇幼卫生事业发展，同时积极争取国际方面的合作与援助。
　　３、“九五”期间，是形成和完善我国健康保障制度的关键时期，要将妇女儿童保健纳入现行职工医疗保健制度改革试点工作；大力推行妇幼保健保偿、合作医疗等多种形式的健康保障制度，提高群众享受妇幼保健和抵御疾病风险的能力。
　　（七）依靠科技进步，努力提高妇幼卫生队伍素质，培养具有良好职业道德和业务素质的专业队伍，是妇幼卫生事业发展的根本保证。“九五”期间，妇幼卫生人才培养的重点是在增加数量的同时，努力提高素质。
　　１、妇幼卫生高等教育要继续发展，进一步改革妇幼卫生教育体制、教学内容和教学方法，走出一条具有中国特色的妇幼卫生专业教育发展道路。要增设妇幼卫生硕士、博士学位导师和培训基地，培养一批高层次专业人才和学科带头人。
　　２、积极推动妇幼卫生、助产等中等教育，加强成人教育和在职教育。
　　３、积极开展农村妇幼卫生人员的培训工作，大力推广适宜技术。
　　（八）建立健全妇幼卫生统计信息网络
　　“九五”期间，建立一支妇幼卫生统计信息专业队伍，形成较为完善的妇幼卫生统计信息网。逐步完善全国孕产妇死亡监测、５岁以下儿童死亡监测、出生缺陷监测体系，不断提高监测水平和对信息的利用能力。逐步建立和完善妇幼卫生统计报表的计算机联网，重视计算机在妇幼卫生工作领域内的使用。
　　（九）普及妇幼卫生健康教育，提高妇女、儿童利用妇幼卫生服务的能力。
　　“九五”期间，要加强妇幼卫生健康教育，利用各种传媒和人际交流等手段，广泛宣传妇幼保健知识；研究和探讨深入社区、深入家庭的有效健康教育方法；开设新婚学校、孕妇学校、育儿学校，把妇幼保健知识宣传到每一个家庭。
　　（十）充分利用外资和国际合作项目
　　总结、推广《加强中国基层妇幼卫生／计划生育服务》项目的做法和经验，发挥项目县的示范、样板作用。“九五”期间，要进一步做好１９９６－－２０００年我国与联合国儿童基金会共同合作的妇幼卫生合作项目和世界银行卫生Ⅵ贷款项目，推动全国贫困地区妇幼卫生工作上新台阶。
　　“九五”期间，要充分利用外资和国际合作，积极争取国际组织、双边合作和民间团体的资金支持和技术援助，进一步打开多渠道、多领域合作的窗口。
　　（十一）建立规范化的妇幼卫生科学管理，使妇幼卫生管理逐步形成法制化、规范化、科学化的运行机制。建立包括信息、决策、评估、反馈等现代化管理的行政管理体制。
　　各级妇幼卫生部门应坚持分级分类的管理原则，针对本地实际情况，针对主要的问题，提出科学的对策，要强化信息反馈和监控，提高工作质量和效率。
　　（十二）大力发展社区妇幼卫生服务。要因地制宜建立和完善社区妇幼卫生服务和管理模式，把妇幼卫生工作做为社区发展的重要组成部分，形成社区政府领导多部门协作配合，全社会参与的局面。
　　（十三）加强职业道德教育，树立高尚的医德
　　要坚持不懈、深入持久的抓好妇幼卫生行业精神文明建设，加强医德医风建设，恪守医德规范，弘扬白求恩精神，全心全意为人民服务。

　　六、实施与评估
　　（一）各级卫生部门要按照本规划纲要的要求，因地制宜，实事求是地制定本地区切实可行的妇幼卫生事业发展“九五”规划和２０１０年目标纲要。要根据卫生部规划纲要所确定的主要目标，争取政府和有关部门的支持，为规划实施提供资金和政策保障。要广泛宣传，动员群众和社会参与，共同实施。
　　（二）根据本规划纲要的目标，建立国家级的报表和监测系统，做好妇幼卫生统计信息的采集、整理、反馈和交流工作，监测本规划纲要的实施情况，为预测发展趋势提供依据。制定切实可行、科学规范的监测评估方案。
　　（三）各级妇幼卫生部门要在各级政府领导下建立定期检查、评审制度。“九五”期间，将进行年度抽样检查和中、终期评估。各地要认真搞好自我评估，以便及时发现问题，采取相应对策，以保证目标按期实现。
c27208--010201xxj

© 北大法宝：（[www.pkulaw.com](https://www.pkulaw.com/)) 专业提供法律信息、法学知识和法律软件领域各类解决方案。北大法宝为您提供丰富的参考资料，正式引用法规条文时请与标准文本核对。
欢迎查看所有[产品和服务](https://www.pkulaw.com/helps/2.html)。法宝快讯：[如何快速找到您需要的检索结果？](https://www.pkulaw.com/helps/70.html)[法宝](https://www.pkulaw.com/helps/69.html)V6有何新特色？

扫描二维码阅读原文

**原文链接：**<https://www.pkulaw.com/chl/4c970f5e2db3c609bdfb.html>
